# Molecular Basis for the Recognition of Adenomatous Polyposis Coli by the Discs Large 1 Protein

**DOI:** 10.1371/journal.pone.0023507

**Published:** 2011-08-17

**Authors:** Zhenyi Zhang, Hua Li, Leyi Chen, Xingyu Lu, Jian Zhang, Ping Xu, Kui Lin, Geng Wu

**Affiliations:** 1 State Key Laboratory of Microbial Metabolism, and School of Life Sciences and Biotechnology, Shanghai Jiao Tong University, Shanghai, China; 2 Key Laboratory of MOE for Developmental Genetics and Neuropsychiatric Diseases, Shanghai Jiao Tong University, Shanghai, China; 3 The Department of Pathophysiology, Key Laboratory of Cell Differentiation and Apoptosis of Chinese Ministry of Education, School of Medicine, Shanghai Jiao Tong University, Shanghai, China; MRC National Institute for Medical Research, United Kingdom

## Abstract

The human Discs Large 1 (DLG1) protein uses two of its three PDZ domains to interact with the C-terminal peptide of the Adenomatous Polyposis Coli (APC) tumor suppressor protein. The DLG1/APC complex inhibits the cell cycle progression from the G0/G1 to the S phase, regulates epithelial cell migration and morphogenesis, and is required for polarization of the microtubule cytoskeleton. However, the molecular details of how DLG1 recognizes APC is not clear. In this study, we performed biochemical and biophysical assays to investigate the interactions between PDZ domains of DLG1 and the C-terminal peptide of APC. In addition, we determined the crystal structures of the PDZ1 and PDZ2 domains of DLG1 each in complex with the C-terminal 11-residue peptide of APC. Our biochemical, biophysical, and structural results revealed structural elements and residues on PDZ1 and PDZ2 domains of DLG1 and on APC crucial for their mutual interaction. In particular, our results show that the β2/β3 loops of PDZ1 and PDZ2 play important roles in contributing to the binding affinities between PDZ domains and APC, through interacting with the residues upstream of the canonical PDZ-binding S/T-X-V motif. The results provide new insights into the binding mode of a defined C-terminal segment of APC by the PDZ domains of DLG1.

## Introduction

**Figure 1:**
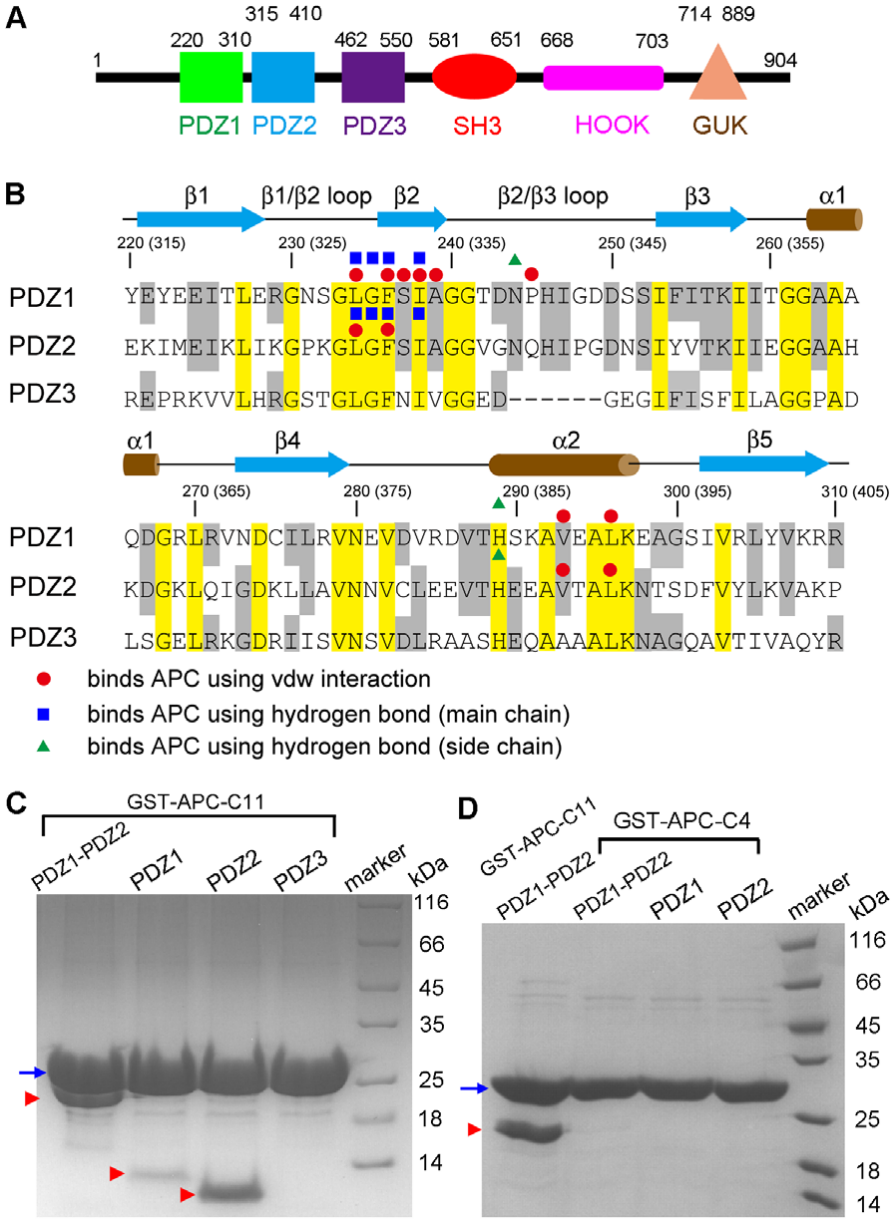
The interaction between the PDZ domains of DLG1 and APC. (**A**) The domain structure of DLG1. The three PDZ domains, the SH3 domain, the HOOK region, and the GUK domain of DLG1 are shown, with their boundary residue numbers indicated. (**B**) Structure-based sequence alignment and secondary structures of the PDZ1, PDZ2, and PDZ3 domains of DLG1. The protein sequences of three PDZ domains were aligned by the ClustalW method. Identical residues in all three PDZ domains are highlighted in yellow, and identical residues in any two PDZ domains are shown in gray. Residue numbers of PDZ1 are shown, and residue numbers of PDZ2 are indicated in parentheses. Residues of PDZ1 and PDZ2 that bind to APC using van der Waals interactions, hydrogen bonding with main chains, and hydrogen bonding with side chains are designated by red circles, blue squares, and green triangles, respectively. (**C**) GST pull-down assay for the interaction between APC-C11 and various PDZ domains of DLG1. Blue arrow indicates the band of GST-APC-C11, and red arrowheads designate the bands of DLG1 PDZ domains. (**D**) GST pull-down assay for the interaction between APC-C4 and various PDZ domains of DLG1. GST pull-down assay between GST-APC-C11 and PDZ1-PDZ2 was also performed in parallel as a comparison. Blue arrow indicates the band of GST-APC-C4 or GST-APC-C11, and red arrowheads designate the bands of DLG1 PDZ domains.

The human Discs Large 1/Synapse Associated Protein 97 (here referred to as DLG1) protein is a member of the membrane-associated guanylate kinase (MAGUK) family, a protein family that has been identified at sites of cell-cell contacts in different organisms including humans [1]. The proteins of the MAGUK family, including DLG1, PSD-95/SAP 90, PSD-93/chapsyn 110, SAP 102, ZO-1, and ZO-2 [2], are multidomain proteins consisting of three PSD-95/Discs Large/ZO1 (PDZ) domains, an Src Homology 3 (SH3) domain, a HOOK region, and a guanylate kinase-like (GUK) domain (Figure 1A). The GUK domains in MAGUK proteins lack key residues required for ATP binding, and it is assumed that guanylate kinase-related regions of these multidomain proteins have adopted a role in protein-protein interactions rather than having an enzymatic function [1]. PDZ domains are made up of ∼90 amino acids and contain a conserved GLGF tetrapeptide motif. They mediate protein-protein interactions by binding to the extreme carboxy terminal S/T-X-V motif of target proteins.

The human tumor suppressor Adenomatous Polyposis Coli (APC) is a 2,843-residue protein. It contains an N-terminal oligomerization domain, an armadillo repeat domain, a central region containing multiple β-catenin-binding and Axin-binding repeats, and a PDZ-binding motif (Thr2841-Ser2842-Val2843) at its extreme C-terminus [3]. The second PDZ domain of DLG1 (residues 315–410, referred to as PDZ2 hereafter) has been reported to bind to APC [4], while neither the first PDZ domain (residues 220–310, PDZ1) nor the third PDZ domain (residues 462–550, PDZ3) of DLG1 has been reported to show substantial APC-binding activity [4]. The APC/DLG1 complex was reported to negatively regulate cell cycle progression from the G0/G1 to the S phase, and over-expression of mutant DLG1 interfered with the cell cycle blocking activity of APC [5]. In addition, it has been shown that APC links microtubules to plasma membranes through interaction with DLG1, so that epithelial cell migration and morphogenesis can be properly regulated [6]. Furthermore, the spatially-localized interaction between APC and DLG1 has been shown to be activated by Cdc42 and the Par6-PKCζ complex at the leading edge of migrating cells, and the interaction between APC and DLG1 is required for polarization of the microtubule cytoskeleton [7].

In addition to APC, several viral proteins implicated in tumorigenesis have also been reported to interact with the PDZ domains of DLG1. For example, the high risk human papillomavirus (HPV) E6 oncoproteins [8], [9], the adenovirus 9 E4 ORF1 protein [8], and human T cell leukemia virus (HTLV) type I Tax protein [10] all contain the S/T-X-V motif, and bind to the PDZ2 domain of DLG1. By binding to the PDZ2 domain of DLG1, these viral oncoproteins might subvert the normal physiological functions of DLG1 during the oncogenic transformation process.

In this study, we investigated the molecular basis of the interaction between DLG1 and APC through a combination of biochemical, biophysical, and structural approaches. We performed the glutathione sulfur transferase (GST) pull-down assay as well as the isothermal titration calorimetry (ITC) assay to analyze the binding between wild-type (WT) or mutant PDZ domains of DLG1 and various C-terminal peptides of APC. In addition, we determined the crystal structure of the PDZ1 domain in complex with the C-terminal 11 residues of APC (APC-C11) as well as the crystal structure of the PDZ2 domain in complex with APC-C11. Both our binding and structural results show that PDZ1 also interacts with APC, though the interaction is weaker than that between PDZ2 and APC. In addition to the canonical binding region, we find that the β2/β3 loops of PDZ1 and PDZ2 contribute to the binding affinity for APC, through interacting with APC residues upstream of the canonical S/T-X-V motif. Our results thus provide new insights into the formation of the DLG1/APC complex and its function.

## Results

### In vitro binding of the C-terminal peptide of APC to the PDZ domains of DLG1

**Figure 2:**
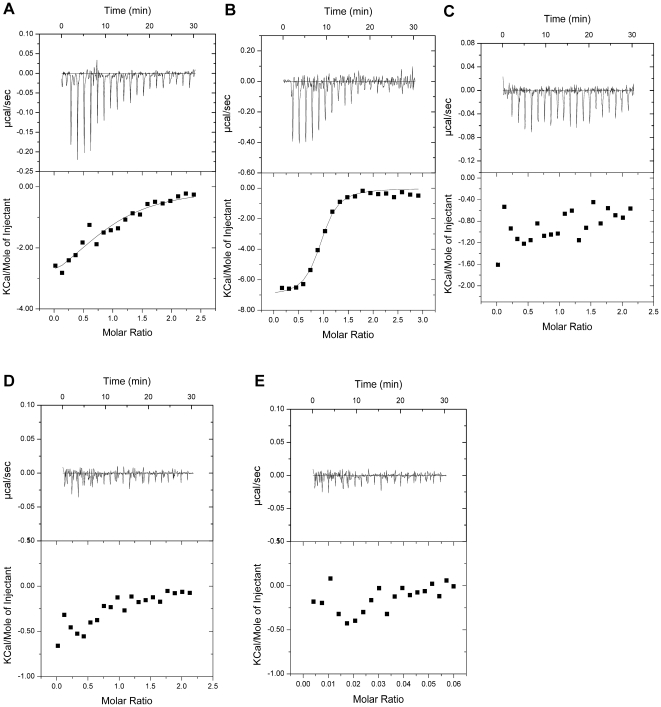
Using the ITC assay to analyze the interactions between various PDZ domains and APC-C11/APC-C4. (**A**) The PDZ1 protein was titrated into the GST-tagged APC-C11 protein, and the dissociation constant (*K*_d_) of the PDZ1/APC-C11 complex was measured to be 18.2 µM. (**B**) The PDZ2 protein was titrated into the GST-APC-C11 protein, and the dissociation constant (*K*_d_) of the PDZ2/APC-C11 complex was measured to be 1.05 µM. (**C**) The PDZ3 protein was titrated into the GST-APC-C11 protein, and no detectable interaction was observed. (**D**) The PDZ1 protein was titrated into the GST-APC-C4 protein, and no detectable interaction was observed. (**E**) The PDZ2 protein was titrated into the GST-APC-C4 protein, and no detectable interaction was observed.

To complement and extend the earlier analysis of the interaction between APC and DLG1, we created two different GST-tagged APC C-terminal peptides: the C-terminal 11 residues of APC (GST-APC-C11: RHSGSYLVTSV), and the C-terminal 4 residues of APC (GST-APC-C4: VTSV). Purified PDZ1, PDZ2, and PDZ3 proteins, which have high pairwise sequence similarities (Figure 1B), as well as the tandem PDZ1-PDZ2 protein construct (residues 220–410), were used to investigate their interaction with GST-tagged APC C-terminal peptides. Both our GST pull-down assay and ITC assay results showed that GST-APC-C11 bound most strongly to the PDZ2 domain (the dissociation constant *K*_d_ = 1.05 µM, Figure 1C, Figure 2B, and Table 1) and to PDZ1-PDZ2 (Figure 1C). Moreover, GST-APC-C11 showed somewhat weaker, but detectable binding to the PDZ1 domain (*K*_d_ = 18.2 µM, Figure 1C, Figure 2A, and Table 1). On the other hand, the binding to APC-C11 was barely detectable for the PDZ3 domain (Figure 1C, Figure 2C, and Table 1), which is consistent with previous findings [4]. Interestingly, the interactions between GST-APC-C4 and PDZ1, PDZ2, and PDZ1-PDZ2 were significantly weaker compared to GST-APC-C11, with no detectable heat of reaction (Figure 1D, Figure 2D, Figure 2E, and Table 1). Therefore no *K*_d_ value could be fit. These results indicate that the canonical S/T-X-V PDZ-binding motif at the C terminus of APC is necessary but not sufficient for efficient binding with PDZ domains. The amino acids upstream of this motif may modulate the binding specificity and affinity. Similar results have also been found for the interaction of NMDA receptor subunits with DLG1, PSD-95, and SAP 102 [11], [12].

**Table 1:** Dissociation constants (*K*_d_) of the interaction between DLG1 PDZ domains and APC-C11/APC-C4 peptides.

DLG	APC	DLG/APC ratio	*K*_d_ (µM)	Δ*H* (kcal/mol)	*T*Δ*S* (kcal/mol)
PDZ1	APC-C11	0.918±0.127	18.2±7.6	−4.02±0.82	2.44
PDZ2	APC-C11	0.916±0.018	1.05±0.22	−7.09±0.19	1.07
PDZ3	APC-C11	n/a	n/a	n/a	n/a
PDZ1	APC-C4	n/a	n/a	n/a	n/a
PDZ2	APC-C4	n/a	n/a	n/a	n/a
PDZ2 Δ339–346	APC-C11	n/a	n/a	n/a	n/a
PDZ2 Q340P	APC-C11	1.04±0.03	9.80±1.11	−6.76±0.27	0.0793

ITC experiments were performed at 25°C. Typically, a 400 µM PDZ domain of DLG1 was injected into a 40 µM GST-tagged APC-C11 or APC-C4 protein solution. “n/a” refers to that no detectable interaction was observed. A negative value for Δ*H* indicates an exothermic reaction while a positive value for Δ*H* indicates an endothermic reaction.

### The β2/β3 loops of PDZ1 and PDZ2 play important roles in the interaction with APC

**Figure 3:**
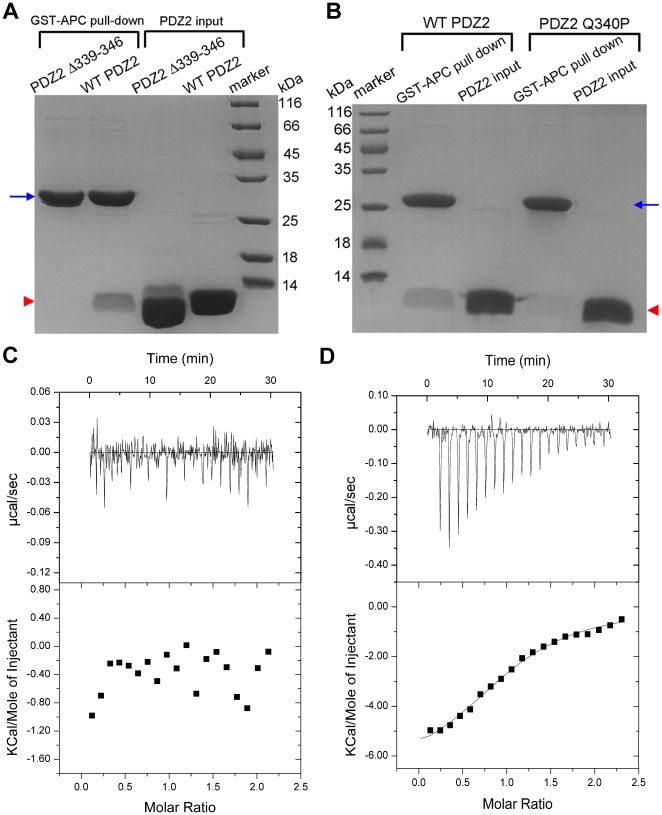
The β2/β3 loop of PDZ2 is important for the interaction of PDZ2 with APC-C11. (**A–B**) Using the GST pull-down assay to analyze the interactions between the mutant PDZ2 domains and APC-C11. (**A**) Deletion of residues 339–346 in PDZ2 dramatically decreased the interaction between PDZ2 and APC-C11. GST-APC-C11 was used for the GST pull-down assay with wild-type (WT) PDZ2 and the mutant of PDZ2 deleting residues 339–346 (Δ339–346). (**B**) Point mutation of Q340P greatly diminished the association between PDZ2 and APC-C11. GST-APC-C11 was used for the GST pull-down assay with WT and Q340P mutant of PDZ2. Blue arrow indicates the band of GST-APC-C11, and red arrowheads designate the bands of WT or mutant PDZ2 domains. (**C–D**) Using the ITC assay to analyze the interactions between the mutant PDZ2 domains and APC-C11. (**C**) The PDZ2 Δ339–346 mutant protein was titrated into the GST-APC-C11 protein, and no detectable interaction was observed. (**D**) The PDZ2 Q340P mutant protein was titrated into the GST-APC-C11 protein, and the dissociation constant (*K*_d_) of the PDZ2-Q340P/APC-C11 complex was measured to be 9.80 µM.

The 3D structures of the three PDZ domains of DLG1 have been determined in the free forms [13]–[15]. They share very similar structures, and the ligand binding pockets are also conserved. Comparison of their amino acid sequences revealed that PDZ1 and PDZ2 have six additional residues in the β2/β3 loops compared to PDZ3 (Figure 1B). It has been reported that deletion of six residues from the β2/β3 loops of the PDZ1 and PDZ2 domains of PSD-95 decreases the binding affinity for the Kv1.4 potassium channel and the NMDA receptor NR2B subunit significantly [16]. The residues in the β2/β3 loops of PDZ domains in other proteins have also been implicated in determining their target ligand binding affinities [17], [18]. In order to know whether the β2/β3 loop also plays an important role in the interaction between APC and DLG1, we created a similar mutant of PDZ2 deleting residues 339–346. Our results showed that deletion of these eight residues from PDZ2 significantly decreases the interaction between PDZ2 and APC-C11, with no detectable heat of reaction (Figure 3A, Figure 3C, and Table 1). The importance of the β2/β3 loop is also supported by the fact that the PDZ3 domain, which possesses high overall sequence homology with PDZ1 and PDZ2 but lacks this loop (Figure 1B), displayed no binding affinity for APC-C11 (Figure 1C, Figure 2C, and Table 1).

Our GST pull-down assay result indicates that the interaction between PDZ2 and APC-C11 is stronger than that between PDZ1 and APC-C11 (Figure 1C, Figure 2A, Figure 2B, and Table 1). A comparison of the amino acid sequence of the β2/β3 loops of PDZ1 and PDZ2 shows that Gln340 in PDZ2 is changed to Pro245 in PDZ1 (Figure 1B). This residue difference might account for the higher affinity between PDZ2 and APC. Indeed, mutation of Q340P leads to substantially weaker interaction between PDZ2 and APC-C11 (Figure 3B). The dissociation constant *K*_d_ value increases from 1.05 µM for the WT PDZ2/APC-C11 complex to 9.80 µM for the PDZ2 Q340P mutant/APC-C11 complex (Figure 2B, Figure 3D, and Table 1). Similar results have also been found for the interaction between the NR2B subunit of NMDA receptors and DLG1, in which the P245Q mutation in PDZ1 led to a 2-fold increase in the binding affinity for NR2B [13].

### Crystal structures of the PDZ1/APC-C11 complex and the PDZ2/APC-C11 complex

In order to obtain detailed structural information on the DLG1/APC interaction, we set up crystallization screens for purified PDZ1/APC-C11, PDZ2/APC-C11 and PDZ1-PDZ2/APC-C11 protein complexes. Unfortunately, attempts to crystallize the PDZ1-PDZ2/APC-C11 complex were unsuccessful, while crystals were successfully obtained for the PDZ1/APC-C11 complex and the PDZ2/APC-C11 complex. The corresponding crystal structures were determined using the method of molecular replacement, to resolutions of 2.30 Å and 2.20 Å, respectively. After refinement, the R-factor/R_free_ was 17.2%/23.2% for the PDZ1/APC-C11 complex, and 20.4%/24.7% for the PDZ2/APC-C11 complex (Table 2).

**Table 2:** Data collection and refinement statistics.

	PDZ1/APC-C11	PDZ2/APC-C11
**Data collection**		
Space group	P3	P21
Wavelength (Å)	0.97916	0.97916
Unit-cell parameters (Å)	a = 105.6, b = 105.6, c = 50.8	a = 67.8, b = 87.4, c = 52.5, β = 102.4°
Number of molecules/asymmetric unit	6	5
Resolution range (Å) (outer shell)	50.0-2.30 (2.38-2.30)	50.0-2.20 (2.28-2.20)
Completeness (%) (outer shell)	99.9 (100)	99.8 (99.9)
Redundancy (outer shell)	5.8 (4.9)	6.0 (6.0)
Total observations/Unique reflections	164,145/28,225	184,827/30,730
R_merge_ (%) (outer shell)	9.9 (35.3)	8.8 (21.6)
I/σ_I_ (outer shell)	16.3 (4.8)	16.8 (7.6)
Wilson B factor	32.9	28.3
**Refinement**		
R_work_ (%)	17.2	20.4
R_free_ (%)	23.2	24.7
Overall B-factor	26.5	38.5
Average B factor for APC-C11	57.7	55.8
Average B factor for the PDZ domain	37.5	38.4
Rmsd bond lengths (Å)	0.009	0.012
Rmsd bond angles (°)	1.253	1.285
Ramachandran plot (most favored, allowed, disallowed, % )	94.4, 5.4, 0.2	96.5, 3.5, 0
Final model (Number of protein atoms)	4,295	3,622
Final model (Number of water molecules)	54	291

R_merge_ = Σ_h_Σ_i_ |*I*_h,i_−*I*_h_|/Σ_h_Σ_i_ *I*_h,i_ for the intensity (*I*) of i observation of reflection h. R factor = Σ||*F*_obs_|−|*F*_calc_||/Σ|*F*_obs_|, where *F*_obs_ and *F*_calc_ are the observed and calculated structure factors, respectively. R_free_ = R factor calculated using 5% of the reflection data chosen randomly and omitted from the start of refinement. Rmsd, root-mean-square deviations from ideal geometry. Data for the highest resolution shell are shown in parentheses.

In the crystal structure of the PDZ1/APC-C11 complex, six complexes can be found in each asymmetric unit. Only one complex was found with electron density for seven residues of APC (residues 2837–2843, SYLVTSV), and the others contain fewer residues of APC. The most complete complex was chosen to analyze the interaction between PDZ1 and APC. In the crystal structure of the PDZ2/APC-C11 complex, four PDZ2 monomers and one PDZ2/APC-C11 heterodimer can be found in each asymmetry unit. Despite considerable effort, only one APC peptide molecule (residues 2838–2843) could be located in complex with PDZ2 (Figure S1). There was no interpretable electron density for the APC peptide near the other four PDZ2 molecules in the asymmetric unit. Compared to the PDZ1/APC complex, the interaction between PDZ2 and APC seems to be weaker because of the low occupancy of APC molecules. This finding is in contrast to our GST pull-down and ITC assay results (Figure 1C, Figure 2A, Figure 2B, and Table 1). We speculate that it is due to the low affinity between PDZ2 and APC-C11 under our crystallization condition. When we performed the GST pull-down assay using a buffer similar to the condition in which PDZ2/APC-C11 crystallized, the interaction between PDZ2 and APC-C11 was also found to be weaker than that between PDZ1 and APC-C11 (data not shown), which was consistent with this speculation.

### The interaction interface between the PDZ1/PDZ2 domains and the APC C-terminal peptide

**Figure 4:**
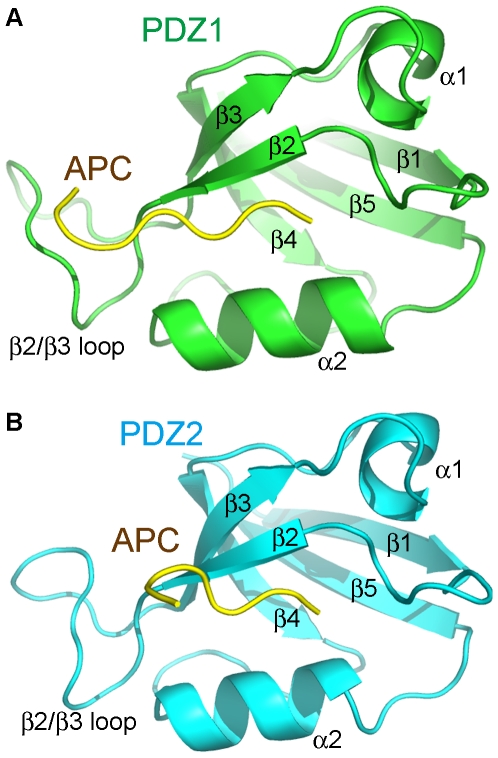
Overall structures of the PDZ1/APC-C11 complex and the PDZ2/APC-C11 complex. (**A**) Overall structure of the PDZ1/APC-C11 complex. PDZ1 is shown in green, while APC-C11 is shown in yellow. Secondary structure elements of PDZ1 are labeled. (**B**) Overall structure of the PDZ2/APC-C11 complex. PDZ2 is shown in cyan, while APC-C11 is shown in yellow. Secondary structure elements of PDZ2 are labeled.

The crystal structures of PDZ1 and PDZ2 are very similar to structures of other typical PDZ domains, each with five β strands and two α helices (Figure 4). As in other structures of PDZ-ligand complexes, Val2843 and Thr2841 of the APC C-terminal peptide are critical for the binding, through interaction with residues on the β2 strand, the α2 helix, and the β1/β2 loop of PDZ1/PDZ2.

**Figure 5:**
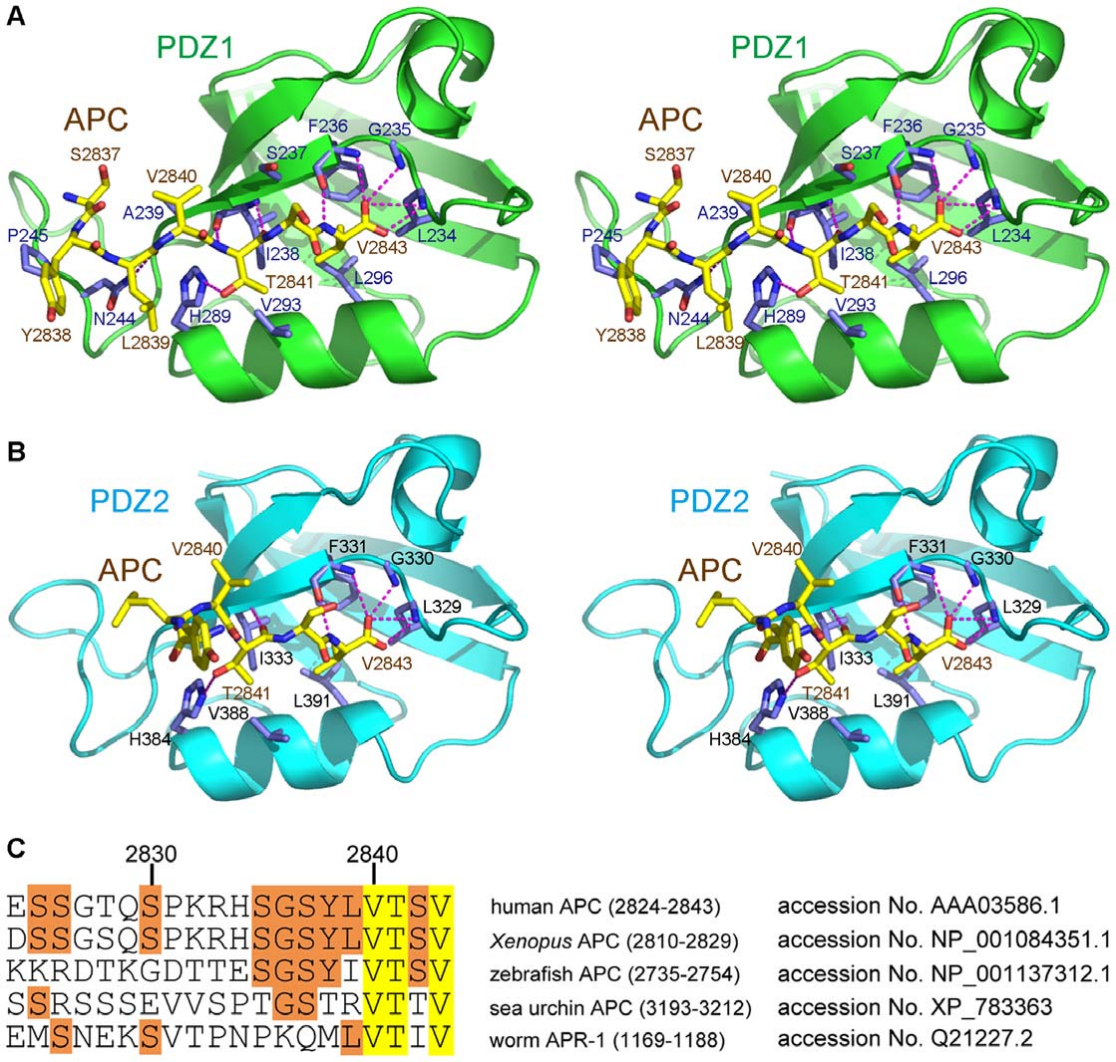
The interaction interface between the PDZ domains of DLG1 and the APC-C11 peptide. (**A**) Close-up stereo view of the interface between PDZ1 and APC-C11 in the crystal structure of the PDZ1/APC-C11 complex. PDZ1 is shown in green, with its side chains shown in purple. APC-C11 is shown in yellow. (**B**) Close-up stereo view of the interface between PDZ2 and APC-C11 in the crystal structure of the PDZ2/APC-C11 complex. PDZ2 is shown in cyan, with its side chains shown in purple. APC-C11 is shown in yellow. Hydrogen bonds are represented by magenta dashed lines. Oxygen and nitrogen atoms are shown as red and blue balls, respectively. (**C**) Sequence alignment of the last 20 residues of APC. Protein sequences of human APC (2834–2843), *Xenopus* APC (2820–2829), zebrafish APC (2745–2754), sea urchin APC (3203–3212), and *C. elegans* APR-1 (1179–1188) were aligned by the ClustalW method. Residues identical among all five homologues are highlighted in yellow, while residues conserved in three out of five homologues are highlighted in orange. Accession numbers for each protein are indicated, and residue numbers for human APC are marked above the sequences.

The most C-terminal residue, APC-Val2843, plays the most important role in mediating the interaction with PDZ1/PDZ2. Its terminal carboxylate group forms hydrogen bonds with main chain amide proton of PDZ1-Leu234/PDZ2-Leu329 and PDZ1-Gly235/PDZ2-Gly330 from the β1/β2 loop, and PDZ1-Phe236/PDZ2-Phe331 from the start of the β2 strand. In addition, the Val2843 side chain is accommodated in a hydrophobic pocket formed by the apolar side chains of PDZ1-Leu234/PDZ2-Leu329 from the β1/β2 loop, PDZ1-Phe236/PDZ2-Phe331 and PDZ1-Ile238/PDZ2-Ile333 from the β2 strand, and PDZ1-Val293/PDZ2-Val388 and PDZ1-Leu296/PDZ2-Leu391 from the α2 helix (Figure 1B, Figure 5A, and Figure 5B). When APC-Val2843 was truncated from the C-terminus of the APC-C11 peptide, no interaction could be observed between the resulting APC peptide and PDZ1/PDZ2 (Figure S2).

APC-Thr2841 is the second most crucial residue for the binding between APC and PDZ1/PDZ2. Its side chain forms an essential hydrogen bond with the imidazole group of PDZ1-His289/PDZ2-His384 from the start of the α2 helix. Moreover, its main chain NH and CO groups form a pair of hydrogen bonds with the backbone of PDZ1-Ile238/PDZ2-Ile333 from the β2 strand. Furthermore, the aliphatic part of its side chain makes van der Waals interaction with PDZ1-Val293/PDZ2-Val388 from the α2 helix (Figure 1B, Figure 5A, and Figure 5B).

Interestingly, in addition to these canonical associations, there are more interactions observed between PDZ1 and APC-C11 in our crystal structure. The N-terminus of the APC-C11 peptide extends towards the groove between the β2/β3 loop and the β2 strand. The β2/β3 loop seems to facilitate the binding of APC by forming interactions with residues upstream of the canonical S/T-X-V PDZ-binding motif. For example, APC-Val2840 forms van der Waals contacts with Ala239 and the aliphatic part of the side chain of Ser237. In addition, the carbonyl oxygen of Leu2839 forms a hydrogen bond with the side chain of Asn244 at the β2/β3 loop, which is conserved in PDZ2. Furthermore, the aliphatic portions of the side chains of APC-Tyr2838 and APC-Ser2837 make van der Waals interactions with Pro245 and Ala239, respectively (Figure 5A). These interactions are observed in most of the six PDZ1 domains in complex with APC-C11 in the asymmetric unit. Notably, the conformations of the β2/β3 loops are also invariable for the different PDZ1 domains (Figure S3).

When the last twenty residues of human, *Xenopus*, zebrafish, sea urchin APC, and *C. elegans* APR-1 are aligned, it is found that the two residues of the canonical S/T-X-V motif, Thr2841 and Val2843, are conserved among all APC homologues. In addition, Ser2835, Gly2836, Ser2837, Tyr2838, Leu2839, as well as Val2840 are conserved among at least three out of the five homologues of APC used for alignment (Figure 5C). The conservation of these residues down to zebrafish and sea urchin underscores the importance of these residues, which are involved in the interaction with the β2/β3 loops of DLG1 PDZ domains (Figure 5A). In contrast, most of the human APC residues before Ser2835 are not as conserved (Figure 5C). The conservation of the DLG1-binding residues in APC suggests that the formation of the DLG1/APC complex might be present among all deuterostomes.

Although we did not observe these non-canonical interactions in the PDZ2/APC-C11 complex structure, it is possible that the crystallization condition we used for producing crystals of the PDZ2/APC-C11 complex was not favorable for these interactions. Most of the residues in PDZ1 mediating non-canonical binding with APC-C11, including Ser237, Ala239, and Asn244, are conserved in PDZ2 (Figure 1B). Therefore, it is reasonable to surmise that these non-canonical associations might also exist between PDZ2 and APC-C11.

### Conformational differences between the free and APC-bound PDZ1 and PDZ2

**Figure 6:**
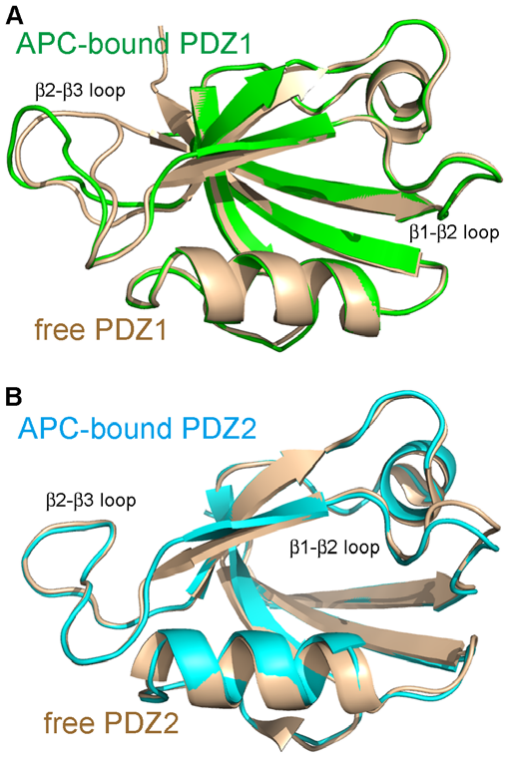
Conformational changes in PDZ1 and PDZ2 upon binding to the APC-C11 peptide. (**A**) The conformational change in PDZ1 upon binding to APC-C11. Structures of free PDZ1 (colored in wheat) and APC-C11-bound PDZ1 (colored in green) were superimposed. (**B**) The conformational change in PDZ2 upon binding to APC-C11. Structures of free PDZ2 (colored in wheat) and APC-C11-bound PDZ2 (colored in cyan) were superimposed. The β1/β2 loops and the β2/β3 loops of PDZ1 and PDZ2 are indicated.

When the structures of free and APC-bound forms of PDZ1 are compared, the conformation of the β2/β3 loop was found to change upon APC binding. The loop moves outward from the core of PDZ1 domain, to create a larger groove between it and the β2 strand (Figure 6A). This movement may facilitate the APC-C11 peptide binding. Although similar movement of the β2/β3 loop was not observed in our PDZ2/APC-C11 complex structure, we suspect that it is because the crystallization condition we used was not favorable for this conformational change, as we discussed earlier. In the structure of the PDZ2/APC-C11 complex, the β1/β2 loop moves slightly away from the α2 helix (Figure 6B), whereas this movement of the β1/β2 loop is not observed in the PDZ1/APC-C11 complex. This movement may be a result of crystal packing, since similar movement can also be found in the free PDZ2 molecules in the same asymmetry unit (Figure S4).

## Discussion

It has been reported that in the free form, the PDZ1 and PDZ2 domains of PSD-95 associate with each other to form a tight interaction [19]. Since the PDZ domains of PSD-95 and DLG1 share very high sequence similarity, analogous interaction between PDZ1 and PDZ2 might also be present in DLG1. Limited proteolysis cleaved the three PDZ domains of DLG1 into two modules. The larger one contained the PDZ1 and PDZ2 domains, suggesting that PDZ1 and PDZ2 interact with each other and together form a protease-resistant module [20]. The solution structure and the crystal structure of PDZ1-PDZ2 of PSD-95 have been solved previously [19], and the solution structure of PDZ1-PDZ2 of PSD-95 in complex with cypin, another protein with the canonical S/T-X-V PDZ-binding motif, has also been determined recently [21]. It has been suggested that cypin-binding induces relative movement between PDZ1 and PDZ2 domains of PSD-95 [21]. Using the structural information of PDZ1-PDZ2 of PSD-95, we can speculate a model of APC-induced inter-domain conformational change of DLG1. In the free form, the β2/β3 loops of PDZ1 and PDZ2 contact each other (Figure S5A). Upon APC binding, the APC-C11 peptides are able to enter the grooves between the β2/β3 loops and the β2 strands of PDZ1 and PDZ2 (Figure S5B). In this situation, the two APC-C11 peptides on both sides push the β2/β3 loops of PDZ1 and PDZ2 towards the middle so that the two β2/β3 loops might collide with each other. Therefore, there has to be a relative movement between PDZ1 and PDZ2 to make a larger space between them and to avoid the collision of the two β2/β3 loops, thus facilitating the binding of APC. In this situation, the inter-domain arrangement of PDZ1-PDZ2 becomes flexible upon APC-binding, which might be the reason that we were not able to obtain crystals of the PDZ1-PDZ2/APC-C11 protein complex.

Although we did not observe the interaction between the β2/β3 loop of PDZ2 and APC-C11 in our structure, our GST pull-down and ITC assay results support that this interaction is critical for PDZ2/APC binding (Figure 3A, Figure 3C, and Table 1). Gln340 in the β2/β3 loop of PDZ2 is crucial for high affinity binding with APC (Figure 3B, Figure 3D, and Table 1), and the P245Q mutation in PDZ1 leads to a 2-fold increase in binding affinity for NR2B [13]. We do not know how Gln340 interacts with APC yet, and alternative crystal forms of PDZ2/APC-C11 are needed to decipher the structural mechanism of how the β2/β3 loop of PDZ2 recognizes APC.

There are two tandem PDZ domains (*i.e.*, PDZ1 and PDZ2) in DLG1, each of which is capable of binding the C-terminal tail of APC. The presence of two APC-binding sites could enhance the binding affinity of DLG1 for APC. Moreover, it is possible that each DLG1 molecule could interact with two APC molecules inside the cell. Interestingly, another APC-interacting protein, end binding protein 1 (EB1), is a homodimer and might also bind two APC [22]–[24]. In addition, APC is reported to be a dimer mediated by the oligomerization domain at its N-terminus [25]. Therefore, it is reasonable to speculate that inside the cell, one APC dimer associates with one EB1 dimer and one DLG1 molecule. Through mediation by EB1, the APC dimer is transported along microtubules while through direction by DLG1, the APC dimer is targeted to the leading edges of migrating cells [7].

APC is an important human tumor suppressor. Mutations in APC are responsible for most of the cases of familial adenomatous polyposis (FAP). DLG1 is the human homolog of the *Drosophila lethal (1) discs large-1* tumor suppressor. The APC/DLG1 protein complex play important roles in the regulation of cell cycle progression [5], cell migration and morphogenesis [6], and cell polarization [7]. In addition, in some cases of FAP patients, mutations of the APC gene (a 4 bp deletion in codon 2644 and a single base deletion in codon 2829) were found close to a site corresponding to the carboxy terminus (codon 2843), the site of DLG1 binding [26]–[29]. Furthermore, several viral oncoproteins, including the high-risk HPV E6 proteins [8], [9], the adenovirus 9 E4 ORF1 protein [8], and the HTLV-1 Tax protein [10], contain the PDZ-binding S/T-X-V motifs in their C-terminus, and have been reported to bind to the PDZ2 domain of DLG1. Therefore, it is possible that disruption of the APC/DLG1 complex, either through mutations in APC or DLG1 genes, or through competitive inhibition by the viral DLG1-binding oncoproteins, might contribute to tumorigenesis.

## Materials and Methods

### Protein Expression and Purification

The cDNAs encoding PDZ1 (residues 220–310), PDZ2 (residues 315–410), PDZ3 (462–552), and PDZ1-PDZ2 (residues 220–410) domains of human DLG1 were cloned into pET28a (Novagen)-derived vectors and over-expressed as N-terminal His-tagged proteins. The C-terminal 11 residues of human APC (residues 2833–2843, APC-C11, with sequence RHSGSYLVTSV), the C-terminal 4 residues of human APC (residues 2840–2843, APC-C4, sequence VTSV), and residues 2833–2842 of APC (APC-C10, sequence RHSGSYLVTS) were cloned into the pGEX4T1 (GE Healthcare) vector to be expressed as N-terminal GST-tagged recombinant proteins.

All proteins except PDZ1-PDZ2 were over-expressed in *E. coli* strain BL21 (DE3), and PDZ1-PDZ2 was over-expressed in *E. coli* strain BL21 (RIL). Cells were cultured at 37°C in LB medium and incubated for 20 hours at 18°C with 0.5 mM isopropyl β-D-1-thiogalactopyranoside (IPTG) on reaching OD_600_ = 0.6. Cells were harvested by centrifugation and resuspended in the binding buffer (Ni^2+^-NTA column binding buffer: 25 mM Tris-HCl, pH 8.0, 300 mM NaCl, and 20 mM imidazole; GST column binding buffer: 25 mM Tris-HCl, pH 8.0, 150 mM NaCl, and 2 mM DTT). The cells were lysed by sonication, followed by centrifugation. Supernatants of the cells lysates were purified by Ni^2+^-NTA affinity chromatography (Qiagen) or GST affinity chromatography (GE Healthcare). PDZ1, PDZ2, PDZ3, and PDZ1-PDZ2 proteins were then purified by Superdex 200 gel filtration chromatography equilibrated with a buffer containing 20 mM Tris-HCl, pH 8.0, 100 mM NaCl, and 2 mM DTT.

The peptide of the C-terminal 11 residues of APC was chemically synthesized, and purified by reverse phase HPLC (Tash Company). The PDZ1/APC-C11 protein complex and the PDZ2/APC-C11 protein complex were prepared by mixing concentrated proteins of PDZ1 or PDZ2 with the APC-C11 peptide, with a molar ratio of 1∶1.5. The final concentration of the PDZ1/APC-C11 complex was 28.4 mg/ml, and the final concentration of the PDZ2/APC-C11 complex was 10.5 mg/ml. The proteins were then flash-frozen in liquid nitrogen and stored in −80°C until use.

### Crystallization and structure determination

Crystals of the PDZ1/APC-C11 complex and the PDZ2/APC-C11 complex were grown at 14°C by the hanging-drop, vapor-diffusion method. Crystals of the PDZ1/APC-C11 complex were grown in 0.1 M HEPES-Na pH 7.5, 0.8 M Na_2_HPO_4_, and 0.9 M KH_2_PO_4_; while crystals of the PDZ2/APC-C11 complex were grown in 0.05 M (NH_4_)_2_SO_4_, 0.1 M Bis-Tris pH 5.5, and 22% PEG3,350. The data sets were collected at the beamline BL17U1 at Shanghai Synchrotron Radiation Facility (China).

Crystals of the PDZ1/APC-C11 complex belonged to the P3 space group, with six PDZ1/APC-C11 complexes in each asymmetry unit. The structure was determined at 2.30 Å, by the method of molecular replacement with the CCP4i program PHASER [30] using the structure of DLG1-PDZ1 by itself (PDB code: 1ZOK) as the search model. The residues of APC-C11 were built into the electron density map with COOT [31]. After refinement with the REFMAC program of CCP4i [30], the model has an R factor of 17.2% and R_free_ of 23.2%. The model quality was checked with the PROCHECK program [30]. The final model includes chain A: residues 220–309 of DLG1, chain B: residues 220–246 and 250–310 of DLG1, chain C: residues 221–310 of DLG1, chain D: residues 220–310 of DLG1, chain E: residues 220–241 and 251–310 of DLG1, chain F: residues 220–309 of DLG1, chain G: residues 2837–2843 of APC, chain H: residues 2838–2842 of APC, chain I: residues 2838–2842 of APC, chain J: residues 2838–2843 of APC, chain K: residues 2839–2843 of APC, and chain L: residues 2839–2842 of APC. In the Ramachandran plot, 94.4%, 5.4%, and 0.2% of residues are in the most favored, allowed, and disallowed regions, respectively.

Crystals of the PDZ2/APC-C11 complex belonged to the P21 space group, with four PDZ2 monomers and one PDZ2/APC-C11 heterodimer in each asymmetry unit. The structure was determined at 2.20 Å resolution using the same method as the structure of the PDZ1/APC-C11 complex. Despite considerable effort, only one APC-C11 molecule could be located in complex with PDZ2 in an asymmetry unit, and there was no observable density for APC-C11 near the other four PDZ2 molecules. Presumably the crystallization condition we used was not favorable for the PDZ2/APC-C11 interaction, and therefore the occupancy of APC-C11 was low in the crystals. After refinement, the model has an R factor of 20.4% and R_free_ of 24.7%. The final model includes chain A: residues 316–409 of DLG1, chain B: residues 316–405 of DLG1, chain C: residues 316–410 of DLG1, chain D: residues 316–409 of DLG1, chain E: residues 316–405 of DLG1, and chain F: residues 2838–2843 of APC. In the Ramachandran plot, 96.5%, 3.5%, and 0% of residues are in the most favored, allowed, and disallowed regions, respectively.

### GST pull-down assays

GST pull-down assays between wild-type (WT) or mutant APC peptides and WT or mutant PDZ domain proteins were performed according to standard procedures. The corresponding fragments of DLG1 were added to GST-APC-C11/APC-C4/APC-C10 protein pre-immobilized on the GST affinity column at 4°C, and then washed using the GST column binding buffer (25 mM Tris-HCl, pH 8.0, 150 mM NaCl, and 2 mM DTT). The bound proteins were eluted with the GST column elution buffer (50 mM Tris-HCl, pH 8.0, 150 mM NaCl, and 7 mM glutathione), and then analyzed by SDS-PAGE and Coomassie Blue staining.

### Isothermal titration calorimetry assays

Isothermal titration calorimetry (ITC) experiments were performed using an ITC200 system (GE Healthcare) at 25°C. The buffer contained 50 mM HEPES, pH 7.5, and 100 mM NaCl. Proteins were centrifuged and degassed before the experiment. Typically, a WT or mutant DLG1 PDZ domain protein (400 µM) was injected 20 times in 2 µl aliquots into a 300 µl sample cell containing GST-tagged APC-C11 or APC-C4 protein (40 µM). Data were fit with a nonlinear least-square routine using a single site binding model with Origin for ITC version 7.0 (MicroCal), varying the stoichiometry (n), the enthalpy of the reaction (Δ*H*), and the association constant (*K*_a_).

### Molecular Graphics

All protein structure figures were generated with PyMOL (http://pymol.sourceforge.net).

### Accession codes

The atomic coordinates and structure factors of the PDZ1/APC-C11 complex and those of the PDZ2/APC-C11 complex have been deposited in the Protein Data Bank with accession numbers 3RL7 and 3RL8, respectively.

## Supporting Information

Figure S1 (TIF)

Figure S2 (TIF)

Figure S3 (TIF)

Figure S4 (TIF)

Figure S5 (TIF)
