# Supplementary figures and images for: Molecular Basis for the Recognition of Adenomatous Polyposis Coli by the Discs Large 1 Protein
Source: PLoS One. 2011 Aug 17;6(8):e23507. doi: 10.1371/journal.pone.0023507 (PMC3157396; doi:10.1371/journal.pone.0023507)

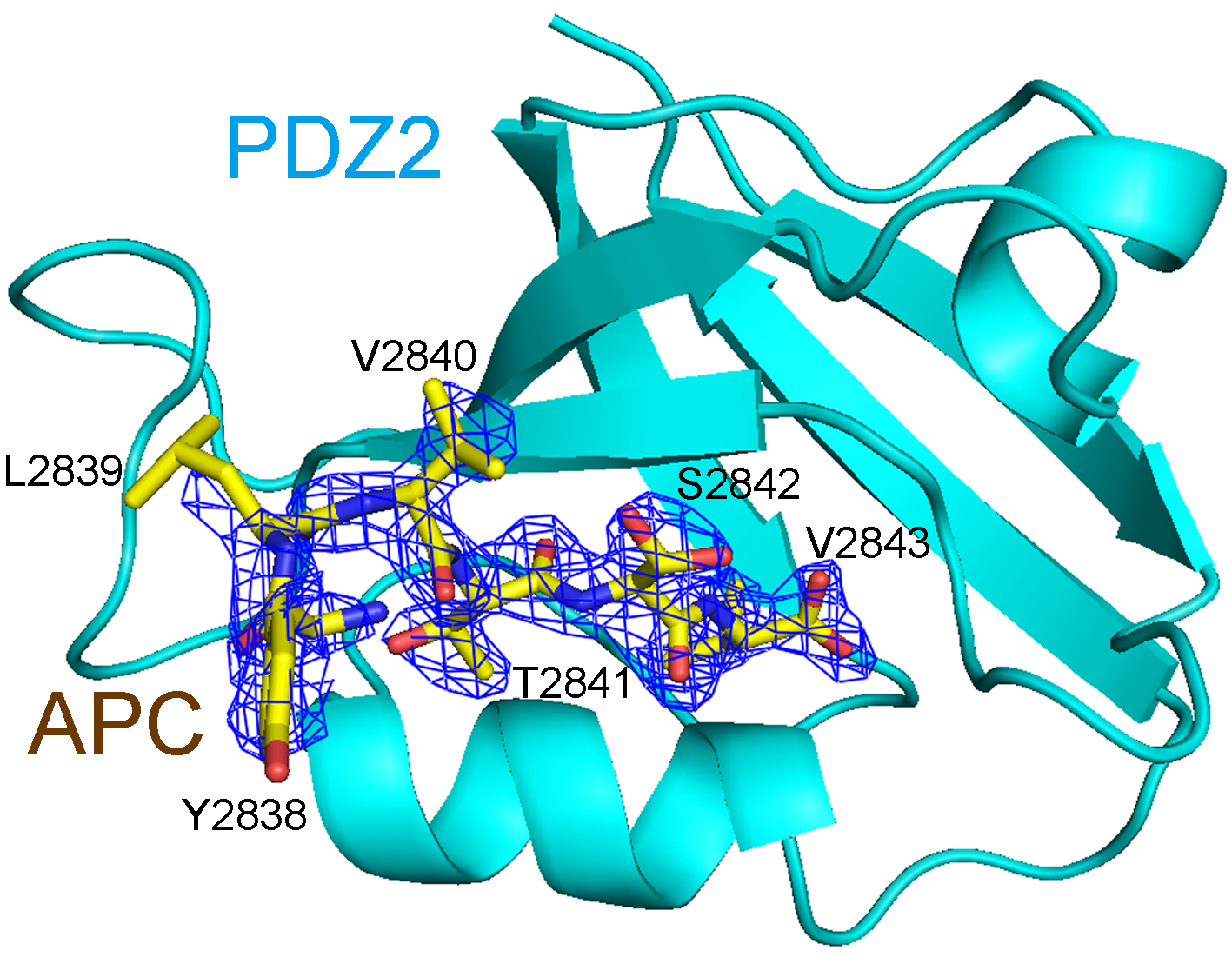

Supplement: Figure S1 — (TIF) [file pone.0023507.s001.tif]

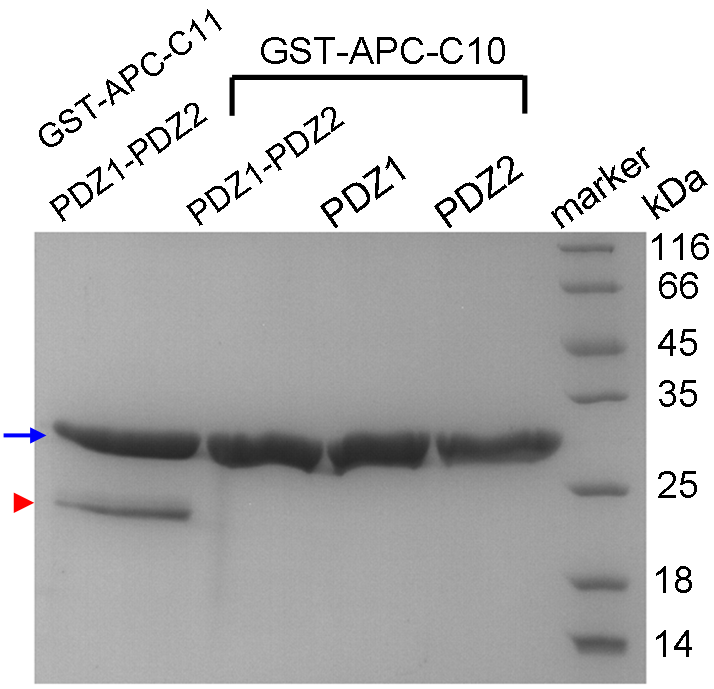

Supplement: Figure S2 — (TIF) [file pone.0023507.s002.tif]

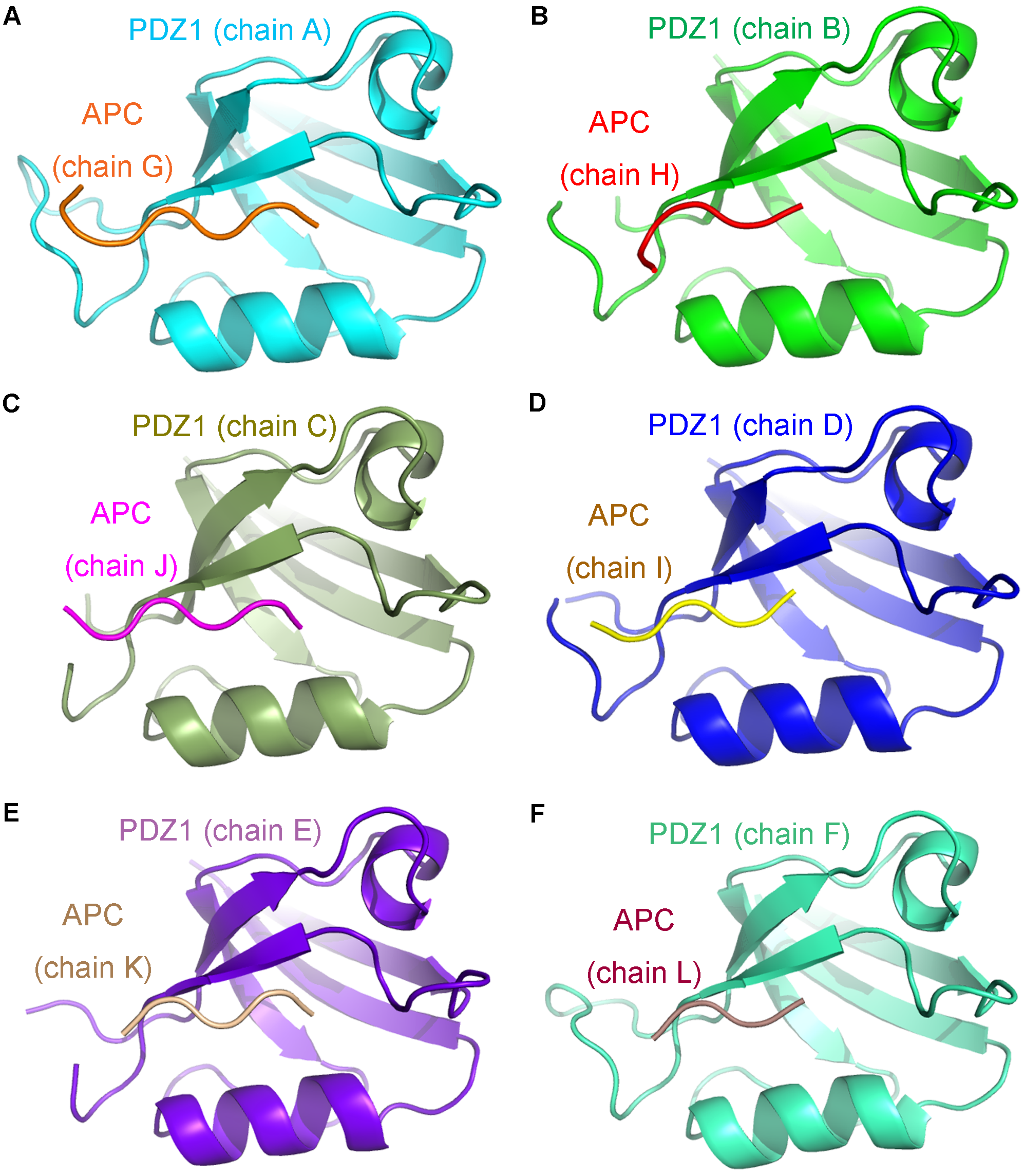

Supplement: Figure S3 — (TIF) [file pone.0023507.s003.tif]

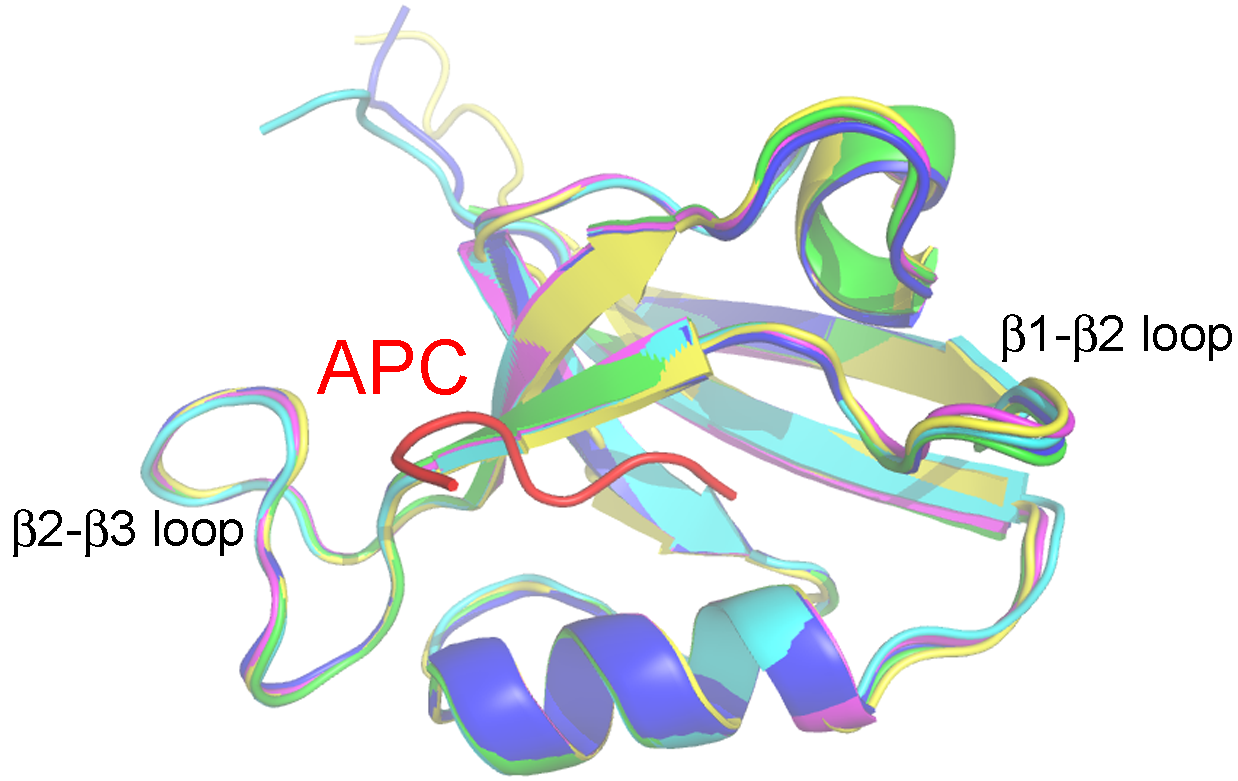

Supplement: Figure S4 — (TIF) [file pone.0023507.s004.tif]

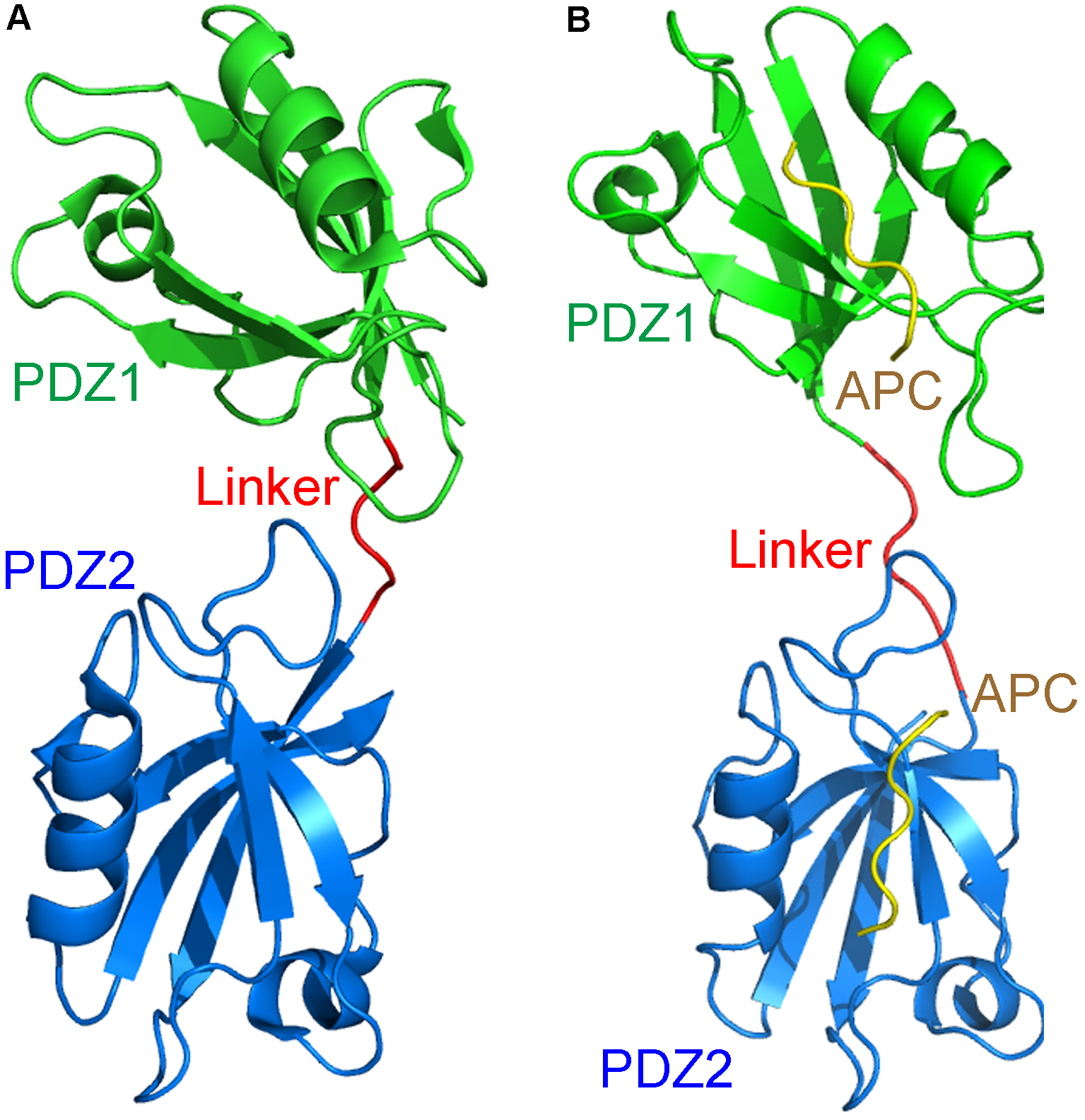

Supplement: Figure S5 — (TIF) [file pone.0023507.s005.tif]
